# Considering the mechanism by which droplets of ALS-FTD-associated *SQSTM1/p62* mutants cause pathology

**DOI:** 10.1080/27694127.2022.2031380

**Published:** 2022-03-17

**Authors:** Yoshinobu Ichimura, Masaaki Komatsu

**Affiliations:** Department of Physiology, Juntendo University Graduate School of Medicine, Bunkyo-ku, Tokyo 113-8421, Japan

**Keywords:** amyotrophic lateral sclerosis, autophagy, liquid droplet, Nrf2, p62

## Abstract

Large numbers of point mutations in *SQSTM1/p62* have been identified in amyotrophic lateral sclerosis (ALS) and frontotemporal degeneration (FTD). SQSTM1 interacts with ubiquitinated proteins, undergoing liquid-liquid phase separation, and the resulting SQSTM1-droplets are degraded by macroautophagy/autophagy. SQSTM1 also serves as a multiple signaling hub for processes including selective autophagy and the anti-oxidative stress response. Such diverse functions are modulated by multiple domains and regions throughout the protein. Because mutations in SQSTM1 have been identified throughout its gene, including regions encoding the domains and motifs, the effects of these mutations on disease onset have been thought to be complicated. Recently, we thoroughly investigated how 7 mutations around the LC3-interacting region and KEAP1-interacting region (amino acids 335-356) affected autophagic degradation of SQSTM1, the anti-oxidative stress response, the KEAP1-NFE2L2/Nrf2 pathway, and the dynamics of SQSTM1 droplets. We found that reduced inner fluidity of the droplets is a unique, shared defect among all mutants, suggesting a link between qualitative changes in SQSTM1 liquid droplets and ALS-FTD. In this punctum article, we discuss the mechanism whereby reduced inner fluidity of mutant SQSTM1 droplets causes ALS-FTD pathology.

## Text

SQSTM1/p62 serves as a receptor protein for selective autophagy against ubiquitinated structures. In fact, this protein localizes to ubiquitin-positive structures such as protein aggregates, damaged organelles, and invading bacteria, and together with such structures is degraded by autophagy. SQSTM1 is also known to be a multifunctional signaling hub, as it participates in apoptosis, the activation of MTOR (mechanistic target of rapamycin kinase) complex 1 in nutrient sensing, NFKB/NF-κB activation during inflammation, and KEAP1-NFE2L2 pathway activation in the anti-oxidative response. In addition, the SQSTM1-mediated NFE2L2 pathway is specifically coupled with selective autophagy. When SQSTM1 interacts with ubiquitinated proteins, they undergo liquid-liquid phase separation and forms SQSTM1 droplets. Thereafter, Ser349 of SQSTM1 in these droplets is phosphorylated, which enhances the binding to KEAP1, an adaptor of the CUL3 (cullin 3)-based ubiquitin ligase for NFE2L2. Thus, KEAP1 is sequestered into the droplets, and NFE2L2 escapes from the KEAP1 interaction and translocates into the nucleus to induce genes encoding proteins that protect against oxidative damage triggered by injury and inflammation. The components of the phase-separated SQSTM1 droplets, including ubiquitin, LC3, and KEAP1, can be exchanged with the surrounding environment. Inside a liquid-like droplet, molecules are predicted to maintain their conformation and activity. Consequently, the droplets can also serve as nodes from which signaling cascades can be activated in the context of selective autophagy and the KEAP1-NFE2L2 pathway. *SQSTM1* has been primarily associated with amyotrophic lateral sclerosis (ALS), frontotemporal lobar degeneration (FTD), and Paget disease of bone. Various SQSTM1-positive structures, including inclusion bodies, have been identified in patients with neurodegenerative diseases such as ALS and FTD. However, the mechanism whereby different mutations throughout the *SQSTM1* gene cause ALS and FTD remains unclear.

Stress granules are liquid-liquid phase-separated droplets that form transiently in the cytoplasm upon specific stress stimuli such as hypoxia, endoplasmic reticulum stress, heat shock, and viral infection. These droplets consist of mRNA, 40S ribosomes, and RNA-binding proteins such as TARDBP/TDP-43 and FUS. The droplets prevent the accumulation of abnormal proteins by transient translation arrest and regulate signaling pathways, thus preventing cellular damage. ALS-FTD-associated TARDBP mutations reduce the phase separation and increase the tendency to aggregate. In addition, it has been reported that droplets consisting of FUS protein change from a liquid to an aggregated state over time, and this propensity is accelerated by patient-derived mutations. These findings suggest a link between stress granule formation or aggregation-transition and the development of ALS-FTD.

Our previous research focused on the formation and properties of ALS-FTD-associated SQSTM1 droplets [1]. Individual SQSTM1 mutants form droplets of varying sizes and numbers in the cells. While the influx rate of cytoplasmic SQSTM1 mutants into SQSTM1 droplets is unchanged relative to wild-type SQSTM1, the droplets composed of these mutants show reduced inner fluidity. Importantly, this reduction is observed in all mutants, regardless of the type of intracellular function that was impaired [1]. The droplets formed by mutations demonstrate droplet aging, a transitional stage from liquid to aggregation. The eventual transition of SQSTM1 droplets to aggregates may lead to cytotoxicity of the SQSTM1 aggregates and functional inhibition of SQSTM1 droplets, resulting in insufficient autophagic degradation of ubiquitinated proteins and a weakened antioxidative stress response due to insufficient activation of NFE2L2.

**Figure 1.**
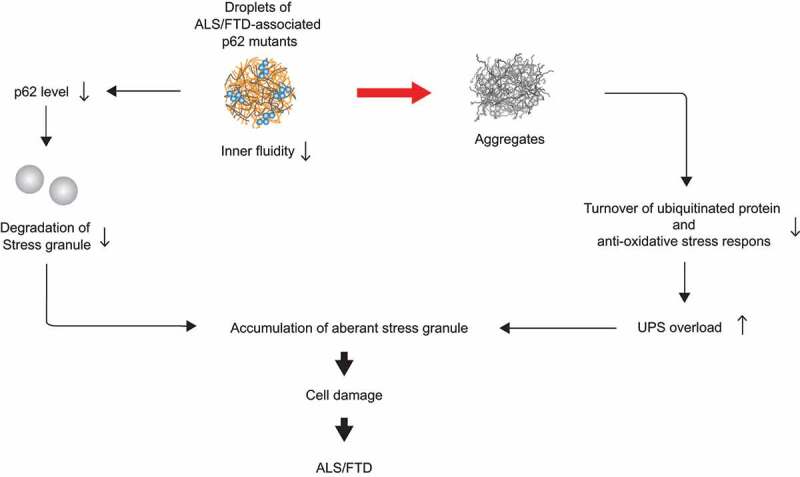
**A model of the mechanism by which droplets of ALS-FTD-associated SQSTM1 mutants cause pathology**. SQSTM1-droplets that exhibit reduced inner fluidity may transit to an aggregated state easily, leading to abnormality in SQSTM1 functions including autophagic turnover of ubiquitinated proteins and antioxidative stress response. Such defects cause the accumulation of aberrant stress granules due to overload to the ubiquitin-proteasome system and impaired turnover of stress granules by autophagy, which may contribute to disease onset of ALS-FTD.

Stress granules and SQSTM1 droplets are considered to be separate structures in cells. Mutations in ALS-FTD-causing proteins such as TARDBP and FUS cause the abnormal formation of stress granules, while mutations in the ALS-FTD-related C9orf72 protein inhibit the removal of stress granules by autophagy. Defective ribosomal products (DRiPs) of stress granules are normally degraded by proteasomes, but stress granules with excessive accumulation of DRiPs are degraded by autophagy. It has been suggested that C9orf72, FUS, SMN (survival of motor neuron), and SQSTM1 are required for autophagic degradation of stress granules, and that defective features of this process are observed in ALS patients. Thus, the reduced inner fluidity of SQSTM1 droplets and their subsequent aggregation may increase the load on the ubiquitin-proteasome pathway by impairing the degradation of ubiquitinated proteins and/or the antioxidative stress response, or hinder the function of SQSTM1 as a selective autophagy receptor, leading to the prevention of stress granule degradation by selective autophagy (Figure 1).

We reported that ALS-FTD-associated SQSTM1 mutants result in qualitative changes in SQSTM1 droplets [1]. However, the following essential issues remain: First, how do different SQSTM1 mutations lead to similar qualitative changes in SQSTM1 droplets? Second, do qualitative changes in SQSTM1 droplets impair stress granule turnover? Third, if SQSTM1 participates in the turnover of stress granules, by what mechanism does SQSTM1 affect their degradation? Fourth, are SQSTM1 mutations involved in motor neuron dysfunction and neuronal death? In order to elucidate these critical issues, several further analyses are required: 1) evaluation of the properties of mutant SQSTM1 droplets such as interfacial tension, viscosity, and elasticity *in vitro*; 2) determination of the protein composition of mutant SQSTM1 droplets in cells; 3) analysis of the dynamics of stress granules and autophagy in cells expressing mutant SQSTM1 droplets; and 4) investigation of the effects of mutants on motor neurons over a long period of time using mutant *Sqstm1* knock-in mice.
